# Experimental analysis and model prediction of elbow pipe's erosion in water-cooled radiator

**DOI:** 10.1038/s41598-024-57174-z

**Published:** 2024-03-22

**Authors:** Yongfei Wang, Xiaofei Li, Tong Wang, Jian Zhang, Longcheng Li, Yu Zhang

**Affiliations:** 1 Chn Energy Dadu River Repair & Installation Co., Ltd., Leshan, 614900 China; 2 Institute of Water Resources and Hydroelectric Engineering, Xi’an University of Technology, Xi’an, 710048 China

**Keywords:** Single-span rotor bearing system, Spindle bending, Spindle crack, Convolutional neural network machine learning, Power stations, Computational science

## Abstract

The radiator with heat transfer capability is able to guarantee the stable operation of hydro generator set, while the long-term and continuous scouring on radiator pipes by cooling medium will lead to thinning or even perforation of pipe wall, which triggers wall failure. This paper analyzes and predicts the failure mechanism of radiator’s pipe wall, and investigates the effects of water flow velocity, sand content and sand particle size on erosion damage of radiator pipe by establishing a test bench for pipe erosion. The results show that the increase of above parameters will lead to the increasing erosion rate, especially when the sand content is 1%, the velocity is 8 m/s and the sand particle size is 0.85 mm, the erosion damage will be particularly serious. Based on experimental data, BP and LSSVM models are employed to predict the pipe wall failure, and PSO algorithm is used to optimize the two models. The optimized PSO-BP has the highest accuracy with the mean absolute error (MAE) of 0.2070 and the mean absolute percentage error (MAPE) of 4.702%. The findings provide a reference for wall failure analysis of radiator, which is of great significance for unit's safe operation.

## Introduction

During the operation of hydro generator set, the high-speed rotation of rotor shaft generates a large amount of heat^1–3^. The accumulation of heat will lead to abnormal heating of unit, damage to equipment and reduced economic efficiency. Radiator is the key equipment to transfer heat, the radiator is installed in bearing oil tank to ensure the safe and stable operation of unit^4,5^. However, as the cooling medium of radiator pipeline is usually taken from river, the impurity-containing water flow will cause erosion damage to pipeline, which will make the radiator functionally ineffective.

There have been some studies on radiators applied to hydro generator sets. Sahel et al^6^. improved the heat transfer efficiency of tubular radiators by optimizing their geometry, and the proposed optimized heat exchanger has the best thermal performance coefficient of 3.58. Dreyer et al^7^. applied temperature sensors with distributed fiber Bragg gratings (FBGs) to bearings and radiators of a hydroelectric generator to closely monitor the radiators' efficiency. Wang et al^8^. pointed out that heat exchangers with Quatrefoil Porous Plate (QPP) structures are a key technology for improving heat transfer performance, and numerical studies have shown that the performance of radiators with this structure has been improved by 27%-41%. Han et al^9^. investigated the expansion strength of radiators with hydraulically expanding connections and experimentally noted that expansion of the joints of piping and fins resulted in more reliable radiator performance. Boukhadia et al^10^. compared the heat transfer performance of a plate-fin heat exchanger with and without baffles, and the results showed that a circular baffle achieves the maximum heat transfer performance coefficient of 2,14. Zhang et al^11^. proposed an optimized scheme for heat-flow coupling of a water-cooled radiator, and stated experimentally that the average surface temperature rise of optimized radiator was reduced by 22.4%. Reviewing the above researches on radiator, most of them focus their attention on structural optimization to improve heat transfer performance or expansion strength, etc.

However, while improving the heat transfer efficiency of the radiator is certainly of interest, attention should also be paid to the effects of cooling medium on heat exchanger itself, such as clogging of pipeline^12^ and perforation of pipe wall^13^. In particular, erosion is the main factor leading to wall perforation. Erosive is mainly influenced by hydrodynamic parameters, particle properties and material properties. It is the most intuitive way to study it by experimentally exploring the erosive wear properties of the material. Researchers have experimentally investigated the erosive behavior of different materials and factors affecting it. Akbar et al^14^. developed a rotary test setup, placed the samples in a slurry environment, and finally compared the microstructure, hardness, and erosive properties of four types of wear-resistant steels. Sarker et al^15^. developed an annular wear tester, and noted that the larger solid diameter leads to the faster settling rate. Li et al^16^. investigated the erosive characteristics of ZTAp / Fe composites and Cr15 at different rotational speeds. Al-Ithari et al^17^. investigated the causes and factors that lead to failures resulting from mechanical wear (erosion) on the internal surfaces of elbows and pipes made from mild steel, and they found ways to reduce such failures. A reduction in the wear rate of elbows and pipes can at least triple their life, thereby reducing maintenance costs by about 75%. Eichner et al^18^. investigated the erosion behavior of aero-engine materials after coating. Kanesan et al^19^. showed that the larger sand particle size leads to greater erosive wear of sand control material based on a jet erosion experimental setup.

It is intuitive to study the erosion phenomenon by experiment, but which cannot predict the service life of eroded pipe and maintenance strategies cannot be further proposed. Pandya et al^20^. modeled erosion using CFD method and machine learning, then proposed a multilayer feedforward artificial neural network. Wang et al^21,22^. developed predictive models of elbows for gas–solid flow conditions with Extreme Learning Machine (ELM), Kernel Extreme Learning Machine (KELM), Hybrid Kernel Extreme Learning Machine (HKELM) and Swarm Intelligence Algorithm (SI). Bahrainian et al^23^. used a novel non-linear method based on Gaussian Regression (GPR) to predict the erosion pattern of solid particles on elbows. Gl et al^24–27^. used decision tree, ANN and Bayesian network, to determine the erosion rate of liquid hydrocarbon pipelines. Zahedi et al^28^. applied a random forest algorithm to predict the erosion rate of a 90° elbow, and the cumulative error of the erosion rate was significantly reduced. Zhu et al^29^. investigated the temperature distribution of erosion bends and proposed a new method for predicting erosion thickness. Using this formula, the thickness of erosion reduction can be calculated simply by monitoring the outer surface temperature of bend. Azhar et al^30^. developed a method to simulate the corrosion behavior of Steel 316L using Artificial Neural Net-works (ANN) and verified the performance of the corrosion modeling by comparing the predicted WT with the actual measurements obtained in experimental tests.

As mentioned above, the existing studies mainly focus their attention on the influence of radiator's material on its life, and various models have been proposed to predict the bending life under specific operating conditions. However, for radiators applied on hydro generator sets, the main factors affecting their tube wall failure are the cooling medium parameters, such as water flow velocity, sand content and sand particle size. Therefore, this paper carries out an experimental study and model prediction of pipe wall failure analysis, and the data of pipe wall erosion rate under the influence of water flow velocity, sand content and sand particle size are obtained by establishing a pipe erosion test bed and carrying out relevant experiments. Furthermore, based on this dataset, BP, LSSVM, PSO-BP and PSO-LSSVM algorithms are employed to predict the pipe wall failure. Which is appiled to achieve the goal of timely warning of pipe wall perforation and failure.

## Test bench for elbow pipe erosion

### Elbow pipe design

**Figure 1:**
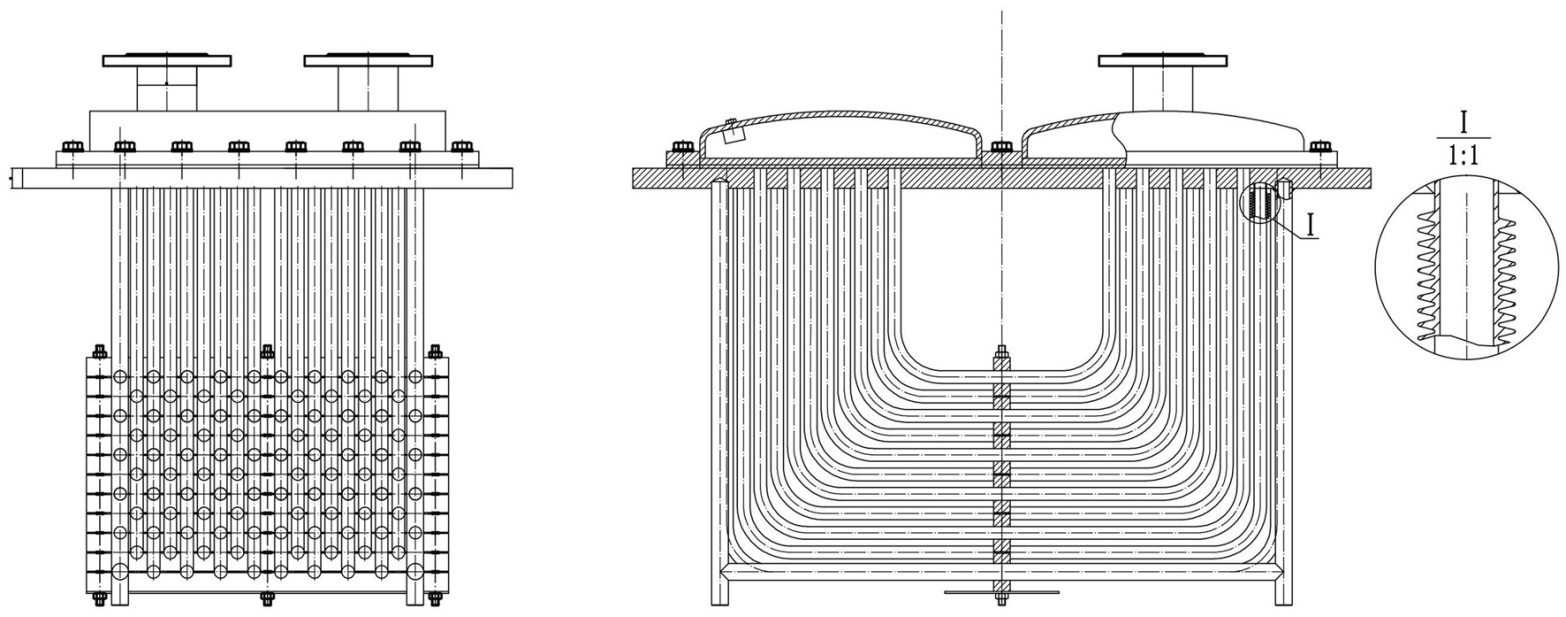
Purple copper, drawer type radiator.

To cool down the bearing of hydroelectric generator sets, most water-cooled radiator adopts the copper drawer type, its structure is shown in Fig. 1. This type of drawer radiator consists of several U-shaped pipes, which is aimed at increasing the heat dissipation area and improving the heat dissipation efficiency. For simulating this bending effect, straight pipe with an inner diameter of 16 mm and a wall thickness of 1.5 mm is selected and machined into 90° bends with a bending radius of 70 mm. Moreover, it is necessary to clean, dry and weigh the manufactured elbow pipes to obtain the original weight, for the purpose of calculating the weight lost by the elbow pipes under erosion.

**Figure 2:**
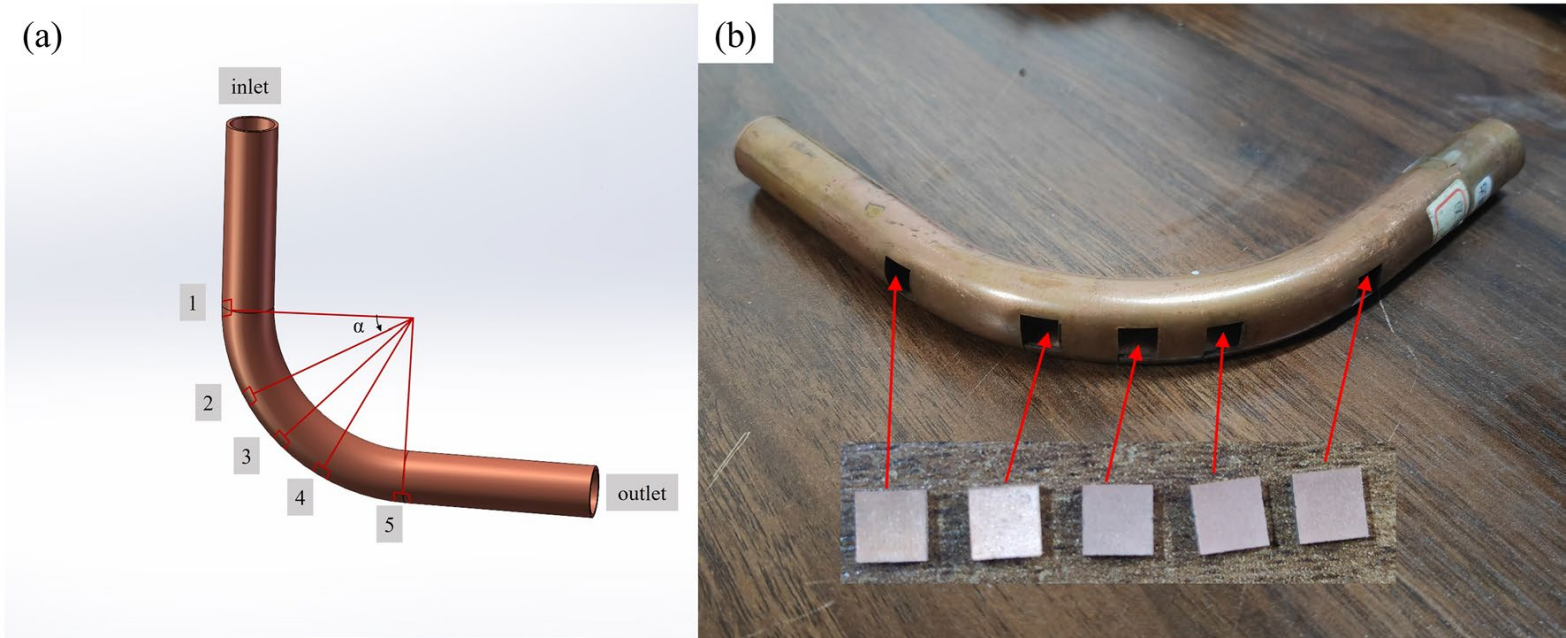
Schematic diagram of different parts of the test piece of bend.

The experimental radiator consists of a straight section and a bent section. And the wear degree of those two section after erosion is different, while the wear behavior is similar. To simplify the analysis of experimental results, only five positions on the outer arch side of the elbow pipe are studied, as shown in Fig. 2. Starting from the pipe’s inlet, numbers 1–5 represent different parts for the outer arch side of pipe specimen, and the studied parts is corresponded to the positions of 0°, 30°, 45°, 60° and 90°, respectively. After erosion experiment, five copper sheets of different parts are obtained by wire cutting for morphological analysis, with a size of 3 cm × 3 cm.

### Experimental setup

**Figure 3:**
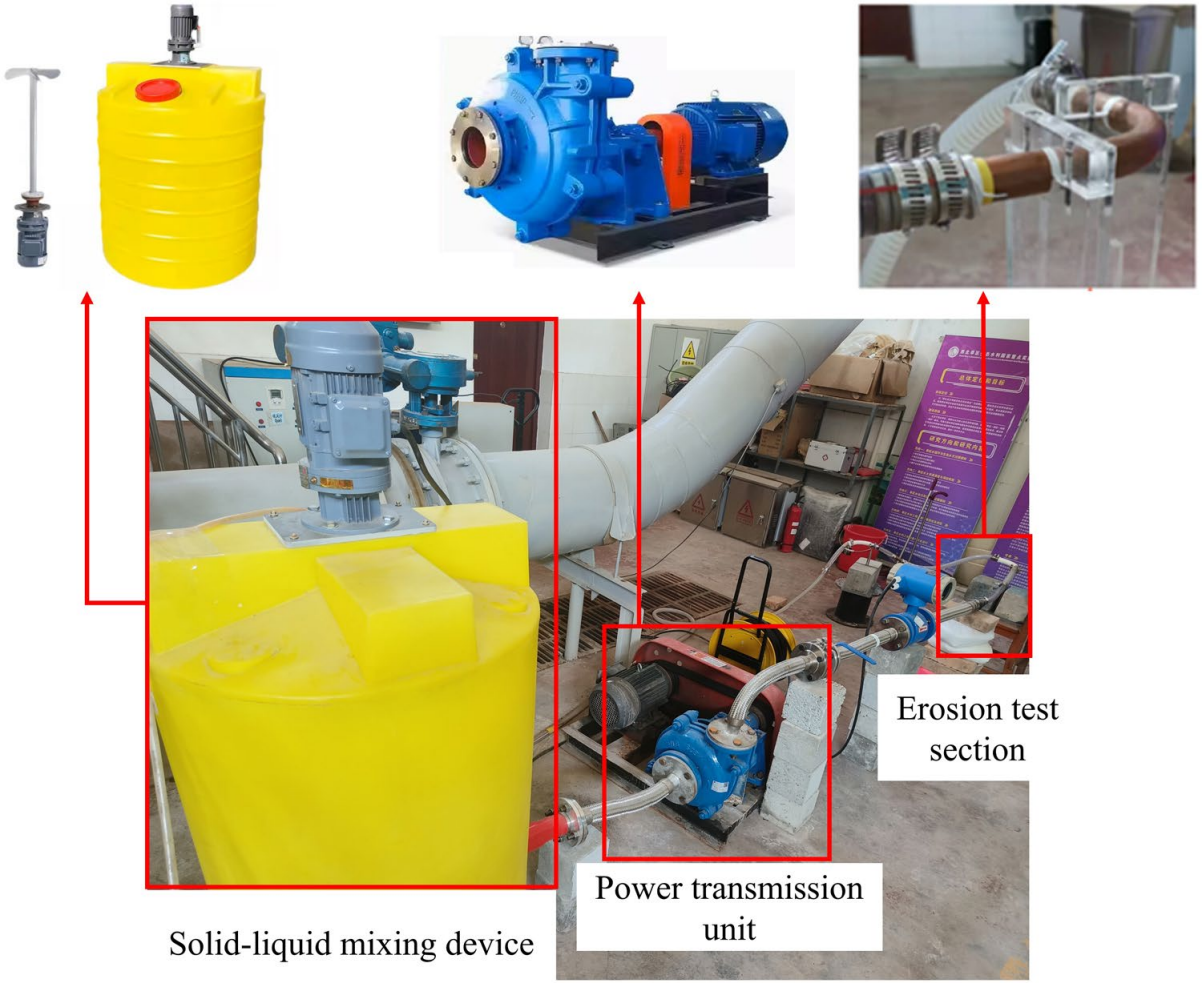
Water-cooled radiator pipe erosion and wear test bench.

The test bench of elbow pipe erosion is built and shown in Fig. 3, which is mainly composed of solid–liquid mixing device, power transmission unit and erosion test section. Solid–liquid mixing device is mainly used to mix water and sand, which includes mixing tank, agitator and etc.; power transmission unit includes slurry pump, bellow and flange ball valve, etc.; erosion test section consists of bending pipe and supporting. Moreover, considering the application of radiators in hydropower units, the cooling medium is generally river water, and the sand contained in different river sections varies, and the selection of a particular river is not representative. Therefore, quartz sand is selected as a solid medium for this test, the hardness of quartz sand is several times that of copper for pipe to ensure that it can impact and destroy the wall in the flow. The main component of quartz sand is SiO_2_, which is mostly in angular form. The range of quartz sand particle sizes used is shown in Table 1.

**Table 1:** Particle size of quartz sand.

Mesh number of quartz sand (Cw)	Range of particle diameter (mm)	Median particle diameter (mm)
20–24	0.8–0.9	0.85
30–35	0.5–0.6	0.55
40–45	0.4–0.45	0.425
70–75	0.2–0.224	0.212

Tap water enters the mixing tank through a pipeline and quartz sand in the required quantity and size is added. The agitator mixes the water with quartz sand to make a two-phase flow. The two-phase flow is pumped into power transmission unit, where the pressure and flow velocity are increased dramatically. High-pressure bellows conveys the pumped two-phase flow to test section. Flow regulation of the two-phase flow is achieved by employing flanged ball valves and an electromagnetic flow meter is used to measure the flow in test section. Elbow pipe is fixed on the supporting to prevent the violent vibration caused by high-pressure and high-velocity flow. Ultimately, the two-phase flow from the test section flows back into the mixing tank to complete its cycle.

### Experimental condition setting

To make the erosion effect obvious, and to save the test material under reasonable conditions, the total time of a single erosion test condition is set to 24 h, and the elbow pipe is weighted every 2 h. Considering the loss of quartz sand after experiment, the two-phase flow is discharged, and the mixing tank is refilled with quartz sand and tap water. Additionally, only the two-phase flow erosive wear on the radiator pipe walls is considered in this work, i.e., the physical damage caused by the impact and scratches on pipe walls resulting from the water containing quartz sand. On the other hand, since the test time is only 24 h, the effects arising from tube wall oxidation in a short period of time are almost negligible. The experimental flow velocity can be obtained from *U* = *Q/A*, where *Q* represents the flow rate measured by electromagnetic flowmeter, and *A* is the cross-sectional area of the pipe.

Before the experiment, the elbow pipe should be cleaned with ethanol, and then weighed three times by electronic balance with an accuracy of 0.1 mg to take the average value, so that the original weight of the specimen before experiments could be recorded. Four types of quartz sand are used, then which are added to the mixing tank and mixed with tap water to make two-phase flow. During the experiment, the supporting and quick release fixture for import and export are removed every 2 h, the elbow pipe is taken out, cleaned, dried and weighed. Meanwhile, to ensure the reliability of test results, the weighing is repeated 3 times, and the average value is taken as the final result. The total erosion time of a test condition is 24 h. Then, the experiment is repeated after changing the experimental conditions (flow velocity, sand content and sand particle size). Finally, the most seriously eroded elbow is selected and the copper sheets are cut at five positions on the outer arch side to observe the surface morphology using an electron microscope. The condition parameters set for the experiment are shown in Table 2.

**Table 2:** Test parameter setting.

Parameter	Value
Bend test piece material	Purple copper
Bend specimen placement method	Horizontal placement
Inlet flow rate (m/s)	2, 4, 6, 8
Particle type	Quartz sand grains
Sand content (wt.%)	0, 0.2, 0.6, 1.0
Sand grain size (mm)	0.212, 0.425, 0.5, 0.85
Contour	Irregularly ribbed
Test time (h)	0–24

The weight changes before and after the test under different working conditions were measured, and the weight loss rate was used to calculate the erosion rate of the specimen, which was calculated by using the formula shown below:

1 $$\Delta \omega = \frac{{m_{0} - m}}{SH}$$

where *m*_*0*_* and m* are the mass of the specimen before and after the test, respectively; Δ*ω* is the erosion rate of the specimen expressed by weight; *S* is the effective overflow area of the specimen; *H* is the erosion time.

## Machine learning fundamentals

### BP neural network

**Figure 4:**
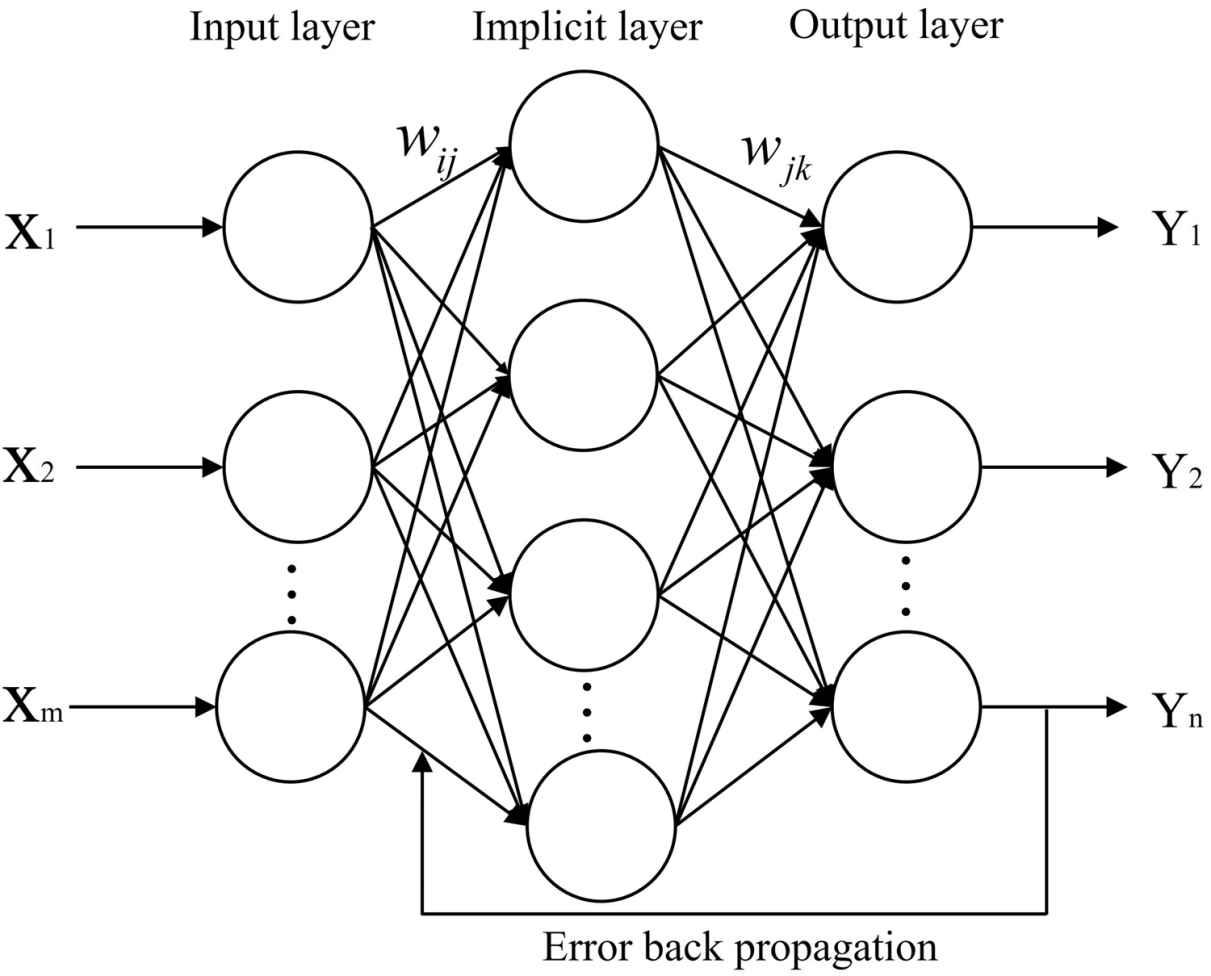
BP neural network structure diagram.

BP neural network is the backward propagation network, which has the characteristics of strong nonlinear mapping ability, strong error tolerance and strong generalization ability. The core of BP is the forward propagation of sample signal and the backward propagation of error. The sample signal starts to propagate from the input layer, then passes between the hidden layers after activation, and finally enters the output layer. The output signal adjusts the weights and thresholds sequentially from the output layer to hidden layer along the direction of smaller error, and if there has been a loss until the predicted output reaches the desired output value or reaches the learning count, the weights and thresholds are adjusted sequentially between the hidden layer andoutput layer. The structure of a typical single hidden layer neural network consists of an input layer, a hidden layer and an output layer, which is shown in Fig. 4.

In Fig. 4, X = (*X*_1_*, X*_2_*, …, X*_m_) and *Y* = (*Y*_1_, *Y*_2_, …, *Y*_m_) are the input and output values of the network, respectively. The training ability of the neural network can be improved by adjusting the weights and thresholds, and usually the number of neurons covered by the hidden layer is determined by specific problem.

The specific modeling process is shown as follows:

The number of nodes *m* in input layer, the number of nodes *l* in hidden layer, the number of nodes *n* in output layer, the learning rate and the activation function are determined from the sequence of samples *(X, Y)*, and the weights *w*_*ij*_, *w*_*ik*_ and thresholds *a*, *b* are initialized. The hidden layer neuron data *H*_*f*_, the predicted output data *O*_*k*_, and the prediction error *e*_*k*_ are computed sequentially:

2 $$H_{f} = f\left( {\sum\nolimits_{i = 1}^{m} {w_{ij} x_{i} + a_{j} } } \right) \, j = 1,2, \cdots ,l$$

3 $$O_{k} = \sum\nolimits_{i = 1}^{l} {H_{i} w_{ik} - b_{k} \, k = 1,2, \cdots ,n}$$

4 $$e_{k} = Y_{k} - O_{k} \, k = 1,2, \cdots ,n$$

where *l* is the number of nodes of the hidden layer neurons;* f* is the hidden layer activation function. The weights *w*_*ij*_、*w*_*ik*_ and thresholds *a* and *b* are adjusted by gradient descent:

5 $$w_{ij} = w_{ij} + \mu H_{j} \left( {1 - H_{j} } \right)x_{i} \sum\nolimits_{i = 1}^{n} {w_{jk} e_{k} \, i = 1,2, \cdots ,m;} \, j = 1,2, \cdots ,l$$

6 $$w_{jk} = w_{jk} + \mu H_{j} e_{k} \, j = 1,2, \cdots ,l; \, k = 1,2, \cdots ,n$$

7 $$a_{j} = a_{j} + \mu H_{j} \left( {1 - H_{j} } \right)\sum\nolimits_{k = 1}^{n} {w_{jk} e_{k} }$$

8 $$b_{k} = b_{k} + e_{k}$$

where *μ* is the learning rate. It is necessary to determine if the algorithm iteration has ended, and if not, the training continues.

### Least squares support vector machine (LSSVM)

Support Vector Machine (SVM) is a supervised method for solving non-linear and high-dimensional problems, which is characterized by global optimization and simple structure^31^. Least Squares Support Vector Machine (LSSVM) is an evolutionary algorithm, which modifies the inequality constraints with equation constraints, and avoids the quadratic regression problem by considering the problem to be solved as a linear programming^32^. The radial basis function is chosen as the kernel function of least squares support vector machine, which is modeled as follows:

A training sample is given by {(*x*_*i*_*, y*_*i*_), *i* = 1, 2*, …, n*}, where *n* is the number of samples. The training samples *φ(x)* are mapped to the high dimensional space by a nonlinear mapping function, and the sample set is shown by

9 $$y_{i} = \omega \cdot \varphi \left( {x_{i} } \right) + b$$

where $\omega$ is the weight vector; *b* is the bias.

Based on the principle of risk minimization, the regression problem is converted into solving the optimization problem:

10 $$\min M\left( {\omega ,\xi } \right) = \frac{{\left\| \omega \right\|^{2} }}{2} + \frac{1}{2}\gamma \sum\limits_{j = 1}^{n} {\xi^{2} }$$

where *ξ* is the training sample regression error variable; *γ* shows the regularization parameter and *ξ*_*i*_ is the bias.

Lagrange functions is introduced to solve optimization problems as follows

11 $$L = \left( {\omega ,b,\xi ,\alpha } \right) = J\left( {\omega ,\xi } \right) - \sum\limits_{i = 1}^{n} {\left( {\omega^{T} \cdot {\text{x}}_{i} + b + \xi_{i} - y_{i} } \right)}$$

### Particle swarm optimization (PSO)

Particle Swarm Optimization (PSO) is a global stochastic optimization algorithm based on population intelligence, which is characterized by high accuracy, few tuning parameters and fast convergence.

The particle is assumed to be searched in D-dimensional target space by a set of particles of size *N*. *X*_*i*_ = (*x*_i1_, *x*_i2_, …, *x*_id_) denotes the position vector of the *i*th particle, and *V*_*i*_ = (*v*_*i*1_, *v*_*i*2_, …, *v*_*id*_) denotes the velocity of the *i*th particle. The single extreme value *p*_*best*_ = (*p*_*i*1_, _pi2_, …, *p*_*id*_) denotes the best position searched by *i*th particle, and the global extreme value *g*_*best*_ = (*p*_*g*1_, *p*_*g*2_, …, *p*_*gd*_) represents the best position searched by *i*th particle in whole target space, expressed as

12 $$V_{id} \left( {t + 1} \right) = w * v_{id} \left( t \right) + c_{1} * r_{1} * \left( {p_{id} - x_{id} \left( t \right)} \right) + c_{2} * r_{2} * \left( {p_{gd} - x_{id} \left( t \right)} \right)$$

13 $$x_{id} \left( {t + 1} \right) = x_{id} \left( t \right) + v_{id} (t + 1)$$

where *v*_*id*_ shows the velocity vector of the *i*th particle after the *d*th iteration; *w* is the inertia weight, whose value is non-negative; *c*_*1*_*, c*_*2*_ are the learning factors of the particles, whose values are in the range of (0,2) interval; *t* is the algorithm after *t* iterations; *r*_*1*_*, r*_*2*_ are the two random numbers between (0,1); and *x*_*id*_ is the position vector of the *i*th particle after the *d*th iteration.

### A. PSO-BP prediction modeling

The BP neural network algorithm has significant advantages such as ease of use and fault tolerance when dealing with multivariate and nonlinear modeling problems, but it is easy to fall into local optimal solutions and slow convergence. So, it is optimized by using the PSO algorithm, which introduces the global search capability of the PSO algorithm^33^. With the introduction of the PSO algorithm, the convergence speed of the BP neural network is accelerated, the efficiency is improved, and the result of local optimization is avoided.

### B. PSO-LSSVM prediction modeling

As for the LSSVM model, its regularization parameter and kernel parameter are significant for the adaptive ability and prediction accuracy. Using of LSSVM to establish the nonlinear relationship between the radiator pipe erosion rate and its influencing factors, improves the model convergence speed and prediction accuracy, and realizes the optimization of the performance of the LSSVM model.

## Experimental results analysis

### Analysis of impact factors

**Figure 5:**
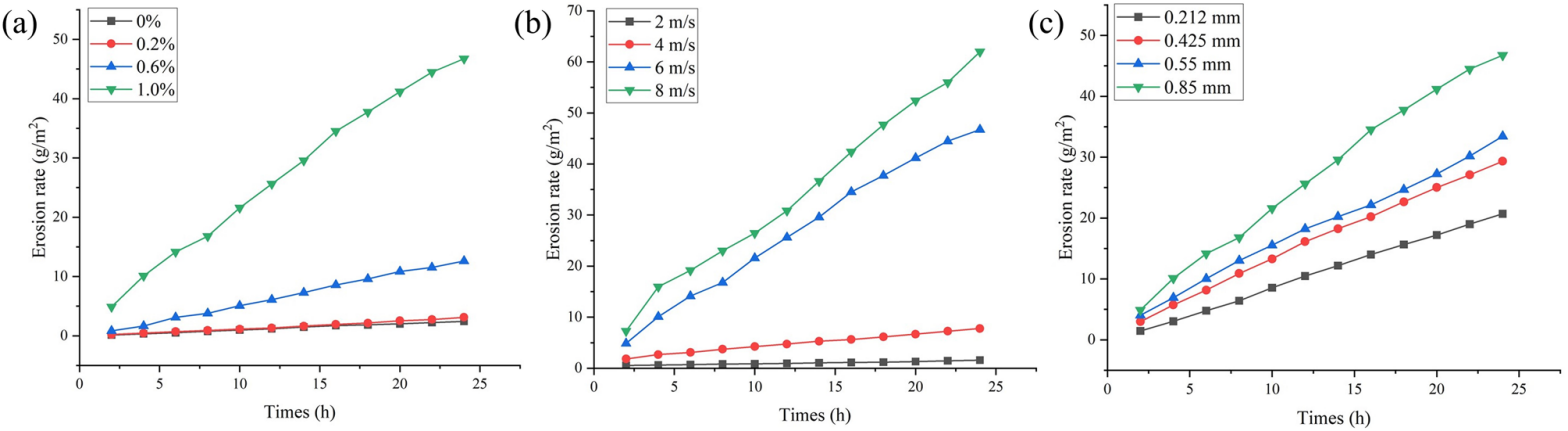
Variation of elbow erosion rate with time affected by different (**a**) sand contents, (**b**) flow rates and (**c**) sand particle sizes.

The experimental erosion rates of the elbow pipe with time under different working conditions are shown in Fig. 5. The influencing factors in Fig. 5(a, b and c) are the sand content, flow velocity of the two-phase flow and sand particle size, respectively. The experimental results show that the weight loss of elbow pipe increases with the increasing erosion time, and the increase of sand content, flow velocity, and sand particle size also accelerates the erosion of bend. Moreover, the flow of water carrying quartz sand inside the radiator's pipes, as a result of the collision between the quartz sand and pipe wall, will continuously lead to the shedding of metal particles from pipe's wall, which causes a decrease in the bend's quality and an increase in erosion rate, and this phenomenon is consistent with the results demonstrated in Fig. 5.

**Figure 6:**
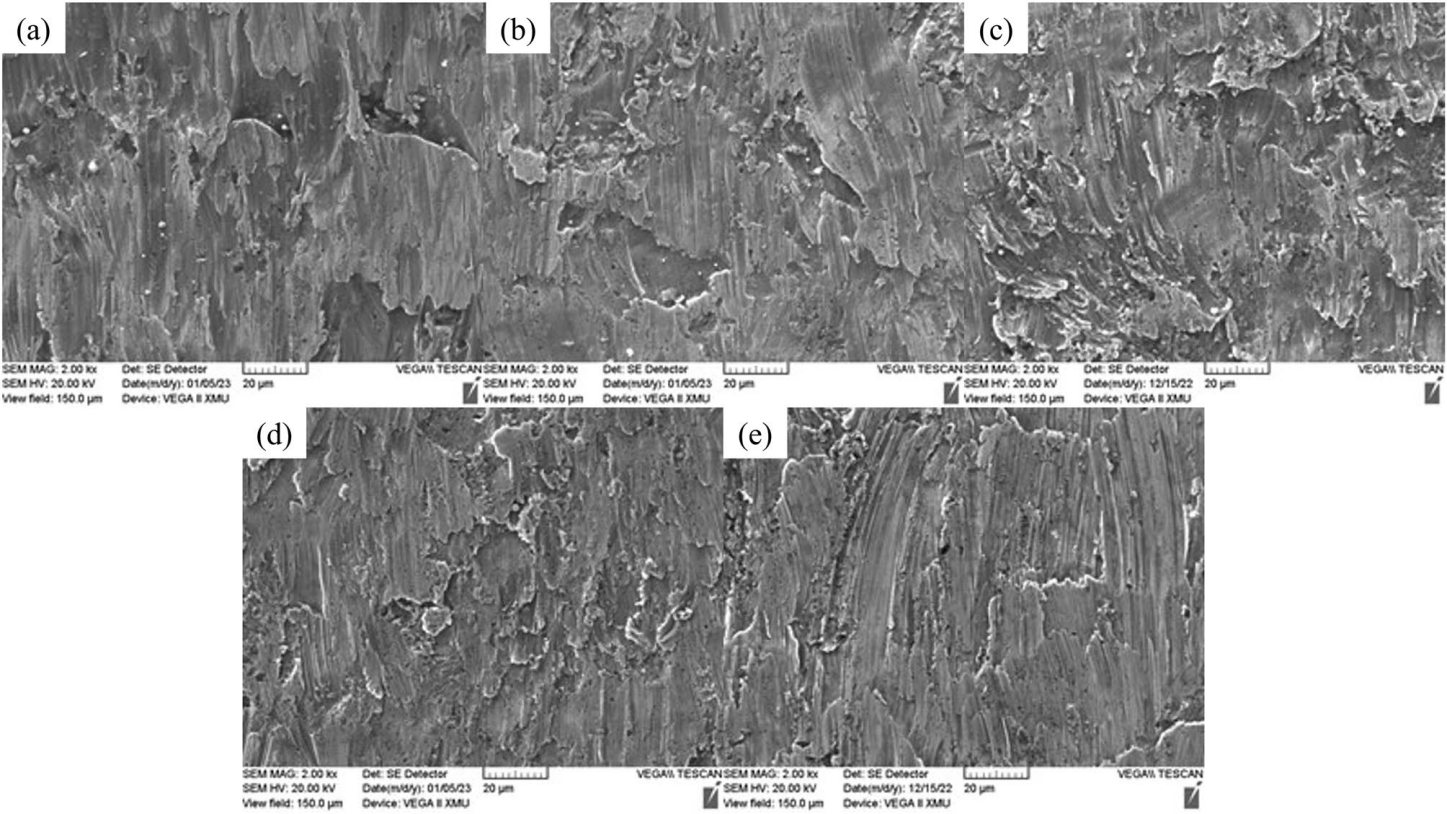
Surface morphology of elbow specimens at different positions of (**a**) 0°, (**b**) 30°, (**c**) 45°, (**d**) 60° and (**e**) 90°.

When erosion occurs in the elbow pipe, because of gravity and centrifugal force, the quartz sand in the two-phase flow will be concentrated to impact the outer arch side of elbow pipe, so the erosion phenomenon occurring here is the most obvious. The increasing sand content, flow velocity and sand particle size will make the erosion more serious. Therefore, the extreme experimental condition (sand particle size of 0.85 mm, sand content of 1.0%, inlet flow velocity of 8 m/s) is selected to test the elbow pipe, and the outer arch side of the elbow pipe as shown in Fig. 2 is cut, then five copper sheets are obtained after the erosion. The surface morphology of copper sheets is observed by employing an electron microscope, as shown in Fig. 6.

The hardness of the irregularly shaped quartz sand is much greater than that of copper, therefore, when the high-pressure, high-speed two-phase flow passes through the elbow, the quartz sand impacts the pipe wall and produces erosion damage to pipes. The tangential force of impact forms grooves and micro-incisions, the normal force directly on the pipe specimen causes extrusion plastic deformation and spalling. Figure 6(a, b, c, d and e) show five copper pieces cut from the outer arch side of the elbow pipe at 0°, 30°, 45°, 60° and 90° positions respectively. Horizontal cuts and grooves can be observed on the surface of specimen at 0°and 30° position, with abrasive build up on both sides of the grooves, as shown in Fig. 6(a,b). Deeper erosion pits and grooves can be viewed from Fig. 6c, which are oriented in the same direction as water flow. The abrasive particles accumulate on both sides and at the end of scratches. The surface morphology of the copper piece at 60°and 90° position is shown in Fig. 6(d,e), where the surface grooves are deeper overall, while the depth of abrasive marks becomes shallow in some areas. And shallow grooves and cuts develop on the surface resulting from the large horizontal component of impact.

Through the above analysis, it can be concluded that the erosion of elbow pipe exists cutting, extrusion, deformation and spalling effects, with the increasing angle, the erosion level is first serious and then reducing, the erosion in outside of the arch at 45° position is the most obvious.

### Model predictions and comparisons

The dataset used for training and validation is derived from the bend erosion test, where sand content, inlet flow velocity, and sand particle size are varied by keeping other conditions unchanged, and the data are recorded every 2 h. 120 sets of test data are recorded. The prediction model is constructed based on the test data and the ordered samples are randomly disrupted, 80% data are taken as the historical data for the establishment of four erosion rate prediction models (BP, PSO-BP, LSSVM, and PSO-LSSVM), and the evaluation of model prediction is carried out for other 20% data shown in Table 3, which is also used to check the accuracy of model.

**Table 3:** Testing set of data.

Sample serial number	Inlet velocity (m/s)	Sand content (wt.%)	Sand particle size (mm)	Erosion time (h)	Erosion rates (g/m^2^)
1	8	1	0.85	8	23.0321
2	6	0	0.85	16	1.6342
3	6	1	0.55	18	23.7928
4	6	1	0.212	6	4.6727
5	6	1	0.55	14	19.4983
6	6	0.2	0.85	16	1.7187
7	8	1	0.85	16	42.4196
8	6	1	0.85	14	28.5176
9	6	1	0.55	2	3.8874
10	6	1	0.212	2	1.4252
11	8	1	0.85	10	0.8613
12	2	1	0.85	16	1.1245
13	2	1	0.85	12	0.9570
14	6	0.6	0.85	4	1.5872
15	4	1	0.85	18	6.1163
16	6	0.2	0.85	14	1.4620
17	6	0	0.85	10	0.9230
18	4	1	0.85	24	7.7685
19	4	1	0.85	8	3.7174
20	6	1	0.425	20	25.3467
21	6	1	0.85	2	4.7017
22	2	1	0.85	24	1.5870
23	6	0.6	0.85	20	10.5296
24	8	1	0.85	24	62.0623

#### Results of BP and PSO-BP prediction model

The fundamentals of BP and PSO-BP are presented in Section "Machine Learning Fundamentals", and it is necessary to further discuss the method of introducing BP by PSO, the fitness and error of PSO-BP. A three-layer BP neural network structure with a single hidden layer is employed, and the number of nodes in hidden layer is directly related to the model training accuracy and training speed, and the trial-and-error method is used to obtain the range of node numbers in hidden layer, which can be calculated by $l = \sqrt {m + n} + a$, where: *l*, *m* and *n* are the node number in hidden layer , input layer and output layer, respectively; and *a* is the adjusting constant with the value interval of 1 ~ 10. The node number in hidden layer is finally set to be 9, so that it has the smallest military error. At this point, the neural network structure is 4–9-1, and the model has a low complexity, and which is sufficient to fit the true rule without matching too much sampling error.

**Figure 7:**
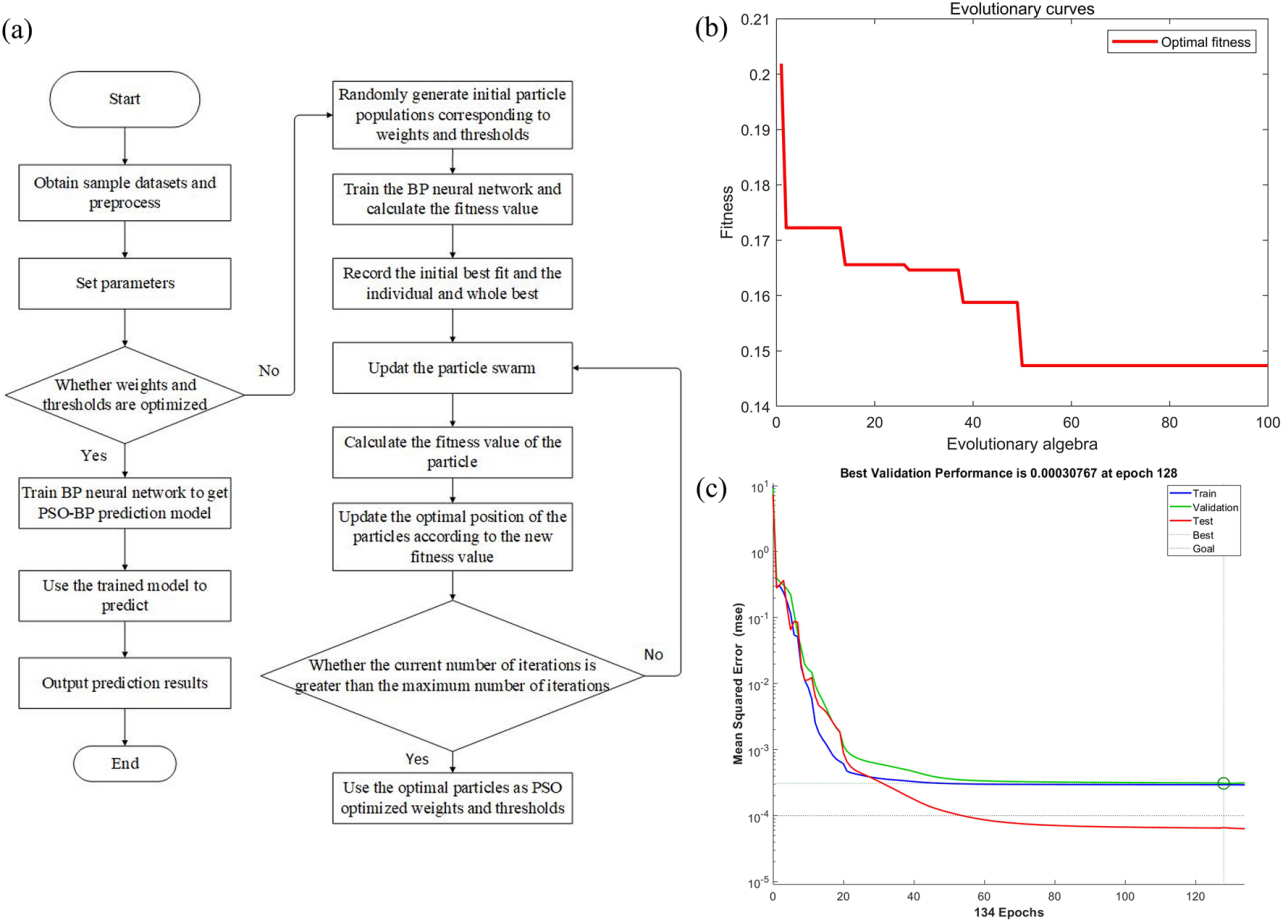
(**a**) Algorithmic logic, (**b**) fitness curves, and (**c**) mean square error for PSO-BP.

The output of PSO is used as the initial weights and thresholds of BP, the best particle position is updated by adaptation value, and the accuracy is improved by iterative optimization. The specific computational flow of PSO-BP model is shown in Fig. 7a. The particle population size selected for PSO is 50, the maximum number of iterations is 100, the learning factor shows *c*_1_ = *c*_2_ = 2, the inertia weight stands for 0.9, and the initialized particles take the velocity in range of [-3,3] and the position in range of [-3,3]. From the 4–9-1 structure of BP, the target space dimension can be determined by *D* = (*m* + *n*)·*l* + *l* + *n*, so *D* takes the value of 55. PSO's fitness function is chosen to be the average of mean square error (MSE) for overall data from training and testing, and the smaller MSE represents the better performance of lattice. Adopt PSO to optimize the parameters of BP, and the model fitness curve is obtained as shown in Fig. 7b.The fitness curve of PSO-BP during the training process decreases rapidly within 50 generations with the smallest MSE, and the convergence reaches the global optimal value. As shown in Fig. 7c, as the number of iterations increases, the MSE of PSO-BP in the training set, validation set and test set decreases continuously, and its prediction accuracy gradually reaches the optimum.

**Figure 8:**
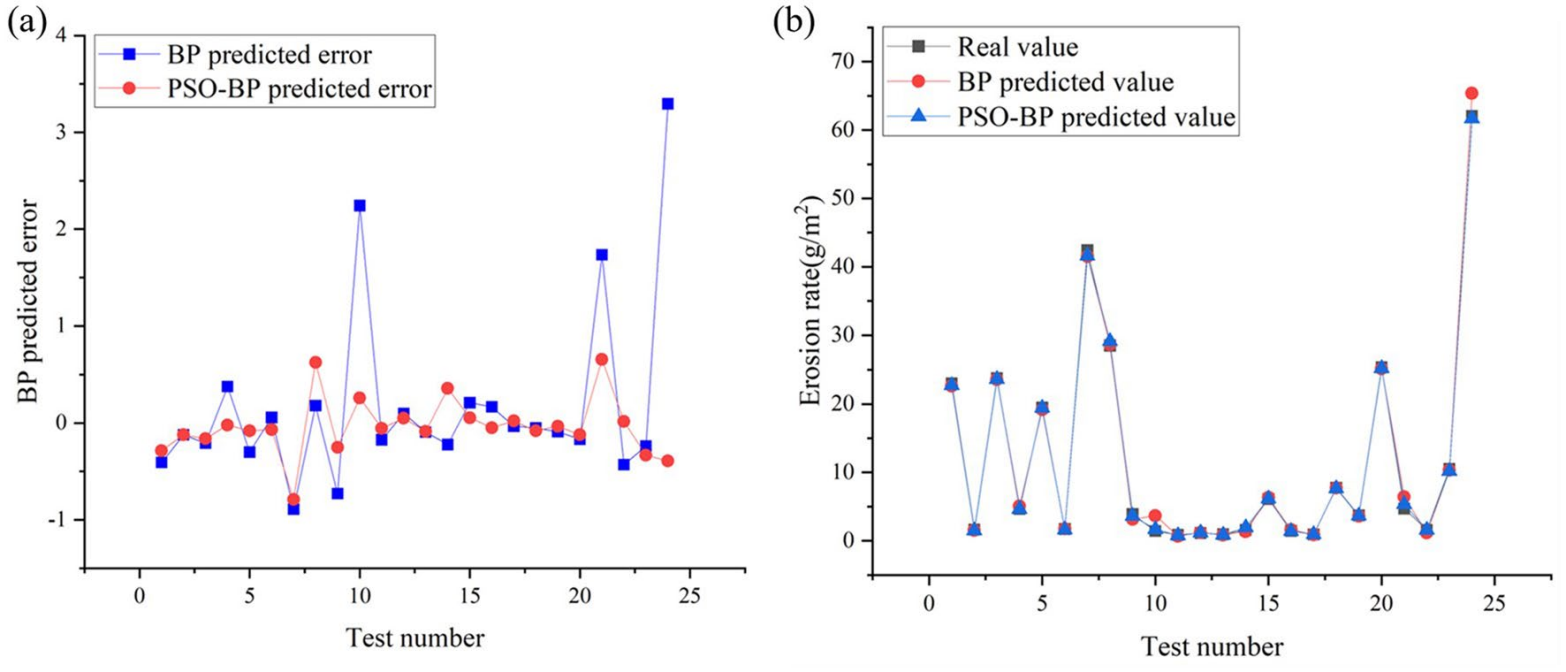
(**a**) Errors, (**b**) comparision of predicted values and tested values of erosion rate for BP and PSO-BP under the test set.

The prediction results of the BP neural network and PSO-BP model are compared with the experimental values to obtain the errors, as shown in Fig. 8a. The error curves evidently show that, the fluctuation of the BP neural network error curve is larger than that of the PSO-BP neural network model, and the maximum value of the prediction error for BP neural network is 3.2945, while the maximum value of the prediction error of PSO-BP neural network is 0.6579. which suggests that, compared with the ordinary BP algorithm, the erosion of radiator elbow pipe can be predicted more accurately by using PSO-BP.

To test the accuracy of the BP and optimized PSO-BP prediction models, based on the 24 working conditions shown in Table 3, the variation of the erosion rate for the elbow pipe with different working conditions is plotted, as shown in Fig. 8b. The results show that the prediction results obtained by the two neural network models are quite close to the experimental erosion rate curves. However, the prediction results of the PSO-BP network model are significantly better than those of BP model.

#### Results of LSSVM and PSO-LSSVM prediction model

The regularization parameters are contained in LSSVM, which reduces the fit of objective function, and the PSO algorithm is used to determine the optimal value of regularization parameter. At the same time, in order to eliminate the computational errors generated by the different magnitudes of erosion influencing factors on model training, and to avoid the generation of singular sample data, the data are normalized to accelerate the convergence speed and prediction accuracy. In this case, each parameter of the selected sample values is normalized into dimensionless quantity to ensure that all data are in the range of [0,1].

**Figure 9:**
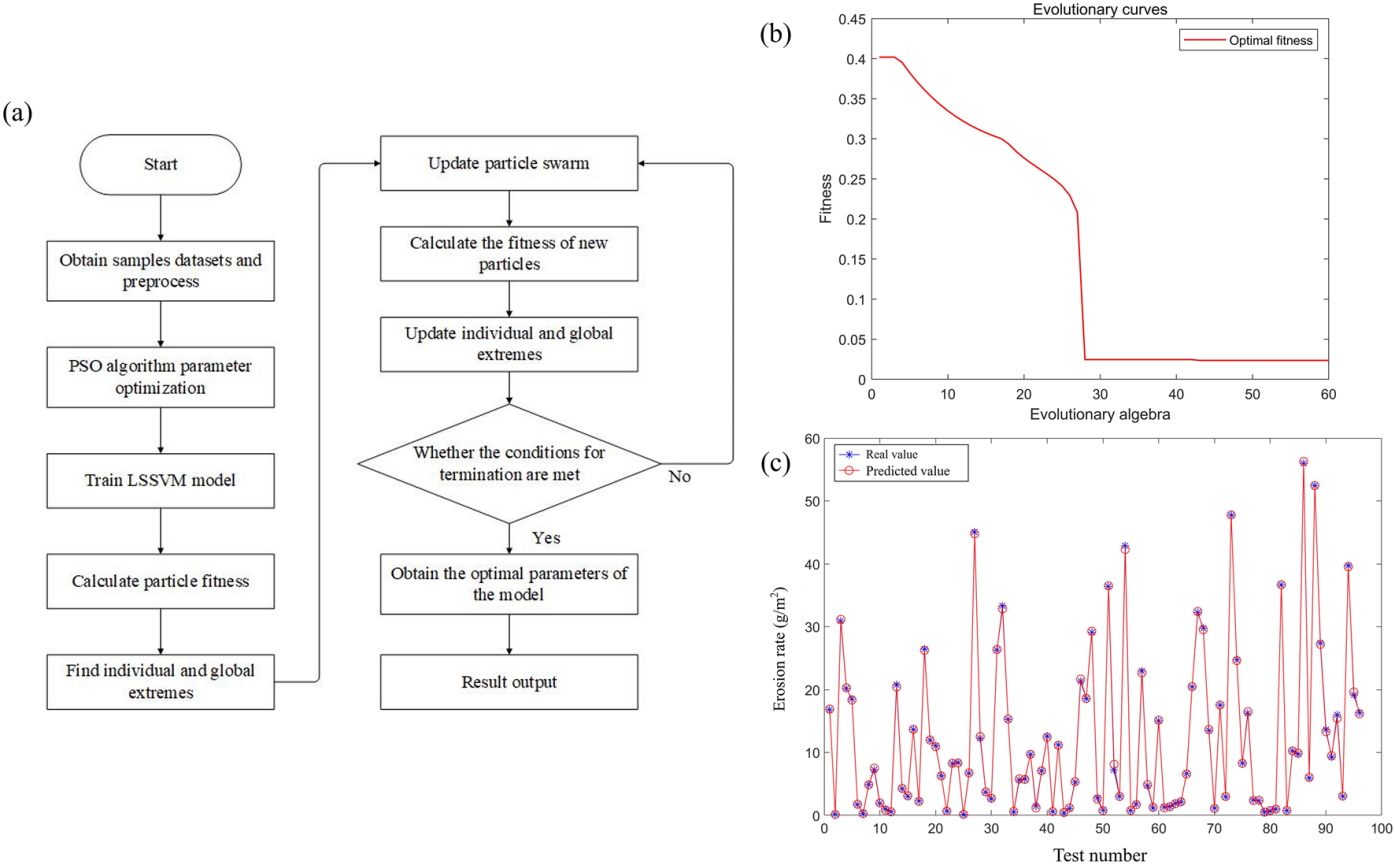
(**a**) Algorithmic logic, (**b**) fitness curves, and (**c**) comparison of predicted and true values of erosion rates for PSO-BP.

The constructed PSO-LSSVM is initialized with each parameter as follows: the spatial dimension and population size are 2 and 30, the maximum number of iterations reaches 80, the learning factor is denoted as *c*_1_ = *c*_2_ = 2, the inertia weight is 0.9, and the regularization parameter γ and the kernel parameter σ take values in the ranges of [0.1,2000] and [0.1,1000], respectively. The resulting combination of γ and σ is trained as the parameters of LSSVM, and the fitness values of particle population for each generation are calculated by the fitness function. Similarly the MSE average of all data from training and testing is chosen as a function to evaluate the particle fitness. The modeling flow of PSO-LSSVM is shown in Fig. 9a. The fitness curve of PSO-LSSVM is shown in Fig. 9b. it can be seen that the MSE decreases rapidly within 28 generations of iterations, and the convergence speed slows down from 28 to 43 generations, and the fitness curve reaches a stable state when the number of iterations amounts to 43 generations, which has a better convergence performance. Furthermore, the prediction of PSO-LSSVM model determined by parameters on the dataset is shown in Fig. 9c. The model prediction and actual erosion rate of all the samples are basically consistent, the error is basically within 0.4, and the vast majority of them are in the range of 0–0.2, which indicates that the model regression fitting accuracy is high, and it can satisfy the actual demand for erosion of radiator pipe.

**Figure 10:**
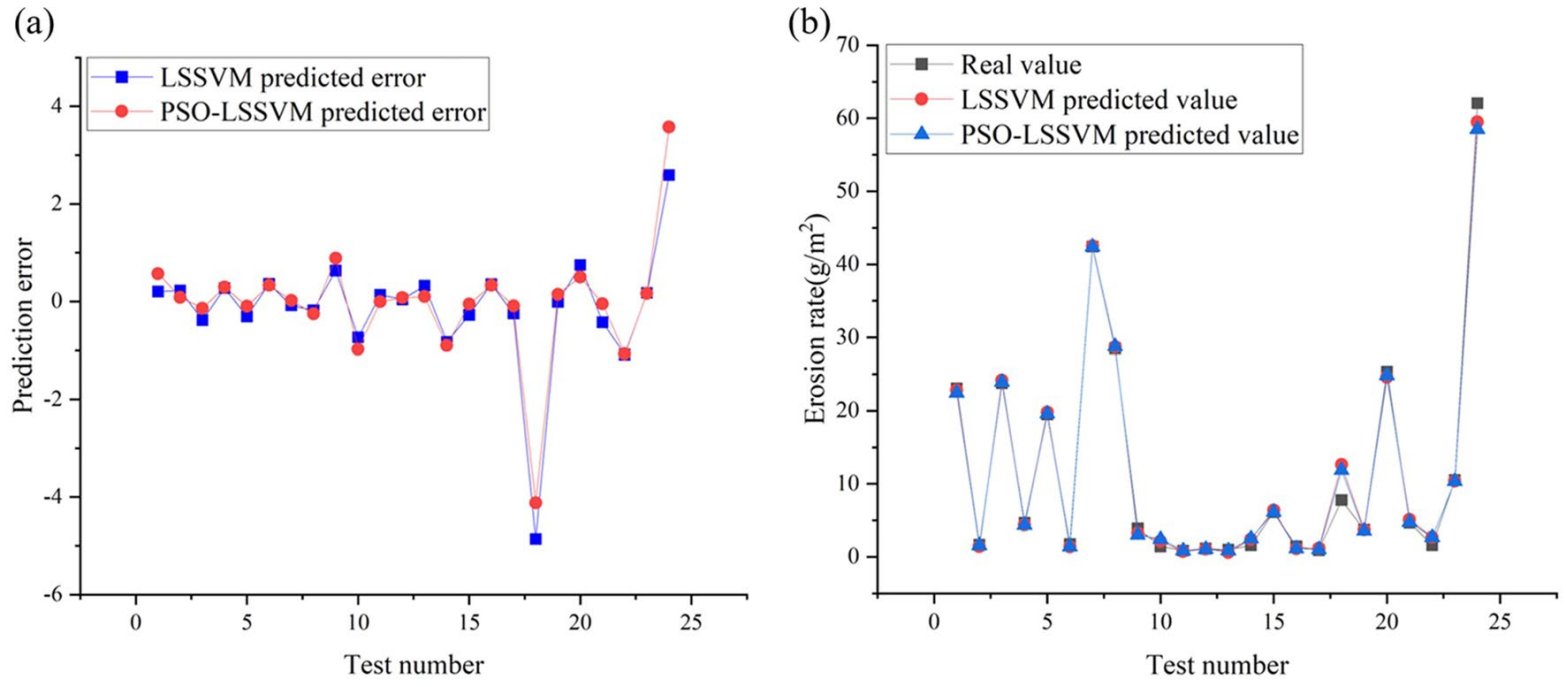
(**a**) Errors, (**b**) comparision of predicted values and tested values of erosion rate for LSSVM and PSO-LSSVM under the test set.

Figure 10a shows the errors of the LSSVM model and PSO-LSSVM model, which are calculated by comparing the experimental data. The results show that the prediction errors of the 24 test samples are basically within 1, and most of them are in the range of 0–0.5. The maximum value of the prediction error for LSSVM model is 3.5740, and the maximum value of the prediction error for PSO-LSSVM model is 2.5917, which indicates that the overall prediction error of the model decreases and the performance of model improves after the optimization of PSO. Similarly, to examine the prediction accuracy and generalization ability of the original LSSVM and the optimized PSO-LSSVM model, based on the experimental results of 24 working conditions as shown in Table 3, the erosion rates of the elbow pipe obtained by the experiment, LSSVM, and PSO-LSSVM under different working conditions are plotted as shown in Fig. 10b. The prediction curves of the PSO-LSSVM model are almost overlapped with those of the experimental curves, which indicates that the effect PSO-LSSVM model is superior and the prediction value is closest to the real value of erosion rate.

#### Comparison of four prediction models

To compare the accuracy of four models more intuitively, MAE, MSE, MAPE and R^2^ evaluation metrics are used to assess the model performance, as shown in Table 4. Among them, MAE, MSE and MAPE represent the mean absolute error, mean square error and mean absolute percentage error, respectively. When values of the three are smaller, the predicted values are more realistic and the accuracy of prediction model is better; the coefficient of determination, R^2^, indicates the correlation between the predicted values and actual values, and when R^2^ is nearer to 1, the accuracy of the prediction model is higher.

**Table 4:** Comparison of prediction errors for the four models.

Predictive model	MAE	MSE	MAPE/%	R^2^
BP	0.5214	0.8816	14.515	0.9969
PSO-BP	0.2070	0.0895	4.702	0.9997
LSSVM	0.6450	1.4528	17.6362	0.9944
PSO-LSSVM	0.6185	1.437	15.3973	0.9950

The results show that, LSSVM has the worst prediction accuracy and the largest fluctuation rate for erosion rate, with a mean absolute error (MAE) of 0.6450 and a mean square error (MSE) of 1.4528. The prediction accuracy of the optimized PSO-LSSVM is improved, however, there is still a gap in the prediction accuracy compared with that of BP neural network. Which is caused by that, LSSVM is more adaptable to small samples. PSO-BP has the highest prediction accuracy for mean square error (MAE) of 0.2070, and the mean absolute error (MSE) of 0.0895 and a mean absolute precision (MAPE) of 4.702%. Meanwhile, the PSO-BP model corresponds to the largest R^2^ of 0.9997, while LSSVM model corresponds to the smallest R^2^ of 0.9944. It can be seen that the correlation between the predicted value and the test value of PSO-BP model is the best, which indicates that the optimized PSO-BP model can accurately predict the erosion of elbow pipe under different working conditions.

## Conclusion

Hydro generator sets produce a large amount of heat during operation caused by the high-speed rotation of rotor, and the accumulation of heat will cause failure and shutdown, which results in equipment damage and huge economic losses. The radiator is the key equipment to take away this heat. This paper explores the effects of water velocity, sand content and sand particle size on pipe's erosion rate of radiator, and the results show that the increase of these parameters will lead to more obvious erosion of the pipe, especially when the sand content of 1%, the flow rate of 8 m/s, the sand particle size of 0.85 mm, the erosion damage will be particularly serious. Further based on these experimental data, BP and LSSVM models are adopted to predict the pipe wall damage, and the PSO algorithm is used to optimize the two models. The optimized PSO-BP had the highest accuracy with a mean absolute error (MAE) of 0.2070 and a mean absolute percentage error (MAPE) of 4.702%.

The purpose of this study is to analyze and predict the thinning, perforation and failure of radiator wall under erosion, which is of great significance for safe and stable operation of the hydro generator set. When carrying out the design of cooler for bearing oil tank on hydro generator set, the cooling medium at radiator inlet can be initially processed so that the water flow rate, sand content, and sand particle size are basically the same as the test conditions, thus the node of pipe wall failure can be roughly predicted by counting the flow time of cooling water in pipeline. Furthermore, the cooling water can be carefully treated to reduce the amount and size of sand, which can slow down the erosion rate and increase the service life of radiator.

Moreover, when the cooling medium contains chemical ions such as ${SO}_{4}^{2-}$, the tube wall undergoes both erosive wear and corrosion damage, which may be closer to the practical application, and under prolonged scouring, the tube wall failure of radiator may be accelerated, which is an interesting and worthwhile phenomenon to be further investigated.

## Data Availability

The datasets generated during and/or analysed during the current study are available from the corresponding author on reasonable request.

## List of symbols

*Q*: Flow in radiator pipe (m^3^/s)
*A*: Cross-sectional area of radiator pipe (m^2^)
*U*: Flow rate of cooling medium in radiator pipe (m/s)
*Δω*: Erosion rate of radiator pipe (g/(m^2^ h))
*m*_*0*_: Initial weight of bend (g)
*m*: Bend weight after erosion (g)
*S*: Effective overflow area of bend (m^2^)
*H*: Erosion time (h)
MSE: Mean square error (–)
MAE: Mean absolute error (–)
MAPE: Mean absolute percentage error (–)
R^2^: Determination coefficient (–)
